# Quantitative Structure–Activity Relationship Modeling and Molecular Docking Studies of TgCDPK1 Inhibitors in *Toxoplasma gondii*

**DOI:** 10.1002/mbo3.70039

**Published:** 2025-07-28

**Authors:** Sara Lesani, Mehdi Tavalla, Gilda Eslami, Mohammad J. Boozhmehrani

**Affiliations:** ^1^ Department of Parasitology and Mycology, School of Medicine Isfahan University of Medical Sciences Isfahan Iran; ^2^ Department of Medical Parasitology, Faculty of Medicine Ahvaz Jundishapur University of Medical Sciences Ahvaz Iran; ^3^ Infectious and Tropical Diseases Research Center, Health Research Institute Ahvaz Jundishapur University of Medical Sciences Ahvaz Iran; ^4^ Student Research Committee Ahvaz Jundishapur University of Medical Sciences Ahvaz Iran

**Keywords:** molecular docking, QSAR modeling, structure–activity relationship, TgCDPK1 inhibitors, toxoplasmosis drug discovery

## Abstract

*Toxoplasma gondii* is a globally prevalent protozoan parasite responsible for severe health complications, particularly in immunocompromised individuals and during congenital infections. Existing treatments are limited by suboptimal efficacy and significant side effects, highlighting the urgent need for novel therapeutic strategies. Calcium‐dependent protein kinase 1 (TgCDPK1) has emerged as a promising drug target due to its critical role in *T. gondii* pathogenesis and structural divergence from human kinases. This study integrates quantitative structure–activity relationship (QSAR) modeling and molecular docking to identify and prioritize potent TgCDPK1 inhibitors. A robust QSAR model was developed from a data set of 152 ligands, leveraging a systematic feature selection process to identify 23 key molecular descriptors predictive of inhibitory activity (*R* = 0.895, *R*² = 0.802). Molecular docking studies further characterized the binding interactions of top‐ranked ligands, revealing strong binding affinities and favorable ADMET profiles. Notably, compound L03, identified as a substituted imidazopyrimidine derivative, demonstrated exceptional binding energy (−176.794 kcal/mol) and stability within the TgCDPK1 active site. Key interactions with Asp210(A) through hydrogen bonds and hydrophobic contacts were instrumental in its high binding affinity, underscoring its potential as a lead compound. These findings provide a comprehensive framework for rational drug design, combining computational approaches to accelerate the discovery of selective and efficacious anti‐toxoplasma agents targeting TgCDPK1. This integrated methodology represents a significant advancement toward addressing the unmet clinical needs of toxoplasmosis treatment.

## Graphical abstract

We computationally identified potent TgCDPK1 inhibitors, a promising drug target for *Toxoplasma gondii*. QSAR modeling using 152 ligands led to IC_50_ predictions with R² = 80.2. Molecular docking prioritized 10 ligands, with the best compound showing strong binding energy (−176.794 kcal/mol), advancing toxoplasmosis drug discovery.

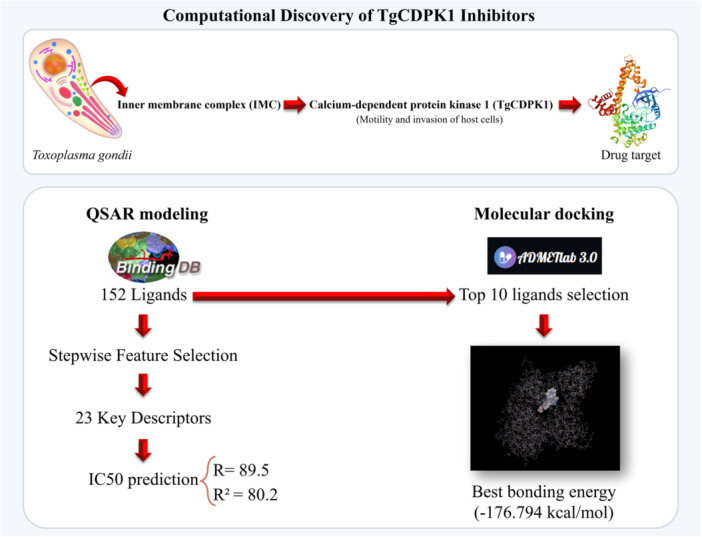

## 1 Introduction

*Toxoplasma gondii*, an obligate intracellular protozoan parasite, poses significant health risks worldwide, infecting nearly one‐third of the global population (Boozhmehrani et al. 2024; Molan et al. 2019). This parasite is notorious for causing severe diseases, including congenital toxoplasmosis and life‐threatening complications in immunocompromised individuals, such as those with HIV/AIDS or undergoing immunosuppressive therapies (Odeniran et al. 2020). Despite the widespread prevalence and clinical impact of *T. gondii*, the therapeutic options remain limited to sulfonamide‐based regimens that exhibit notable drawbacks, including significant side effects and limited efficacy against chronic stages of the infection (Arafa et al. 2023). Additionally, the absence of an effective vaccine underscores the urgent need for novel therapeutic interventions (Innes et al. 2019).

Among the molecular targets explored in *T. gondii*, protein kinases have emerged as promising candidates due to their essential roles in parasite survival and pathogenesis (Dos Santos et al. 2023). These kinases, particularly calcium‐dependent protein kinase 1 (TgCDPK1), are crucial for processes such as host cell invasion, motility, and egress (Wei et al. 2013). TgCDPK1's unique structural features, which distinguish it from mammalian kinases, make it an attractive target for selective drug development (Wei et al. 2013). Recent advancements have identified potent inhibitors of TgCDPK1, demonstrating the feasibility of targeting this kinase for antitoxoplasmosis therapy (Jin et al. 2022; Yang and Sun 2024; Gharibi et al. 2023).

Quantitative structure–activity relationship (QSAR) modeling is a computational approach that has proven instrumental in drug discovery, enabling the prediction of biological activity based on molecular descriptors (Shah et al. 2024).

Previous studies have strongly underscored the potential of TgCDPK1 as a selective and essential therapeutic target in *T. gondii*. For example, ATP‐competitive inhibitors designed against TgCDPK1 demonstrated high selectivity over human kinases due to the enzyme's unique glycine gatekeeper residue, and effectively blocked parasite invasion and proliferation without affecting human cell lines (Johnson et al. 2012). Beyond direct antiparasitic effects, TgCDPK1 has also been implicated in *T. gondii*‐induced neuropathogenesis; a recent study showed that ginsenoside Rh2 (GRh2) exerts neuroprotective effects by binding to TgCDPK1 and suppressing the NLRP3 inflammasome signaling pathway in microglia (Jin et al. 2022). Additionally, DNA vaccines encoding TgCDPK1 have been shown to induce strong humoral and cellular immunity in mouse models, further validating TgCDPK1 as a viable immunotherapeutic target (Chen et al. 2014). Despite these advances, the application of QSAR modeling to TgCDPK1 inhibition remains relatively limited. Although a 3D‐QSAR and docking‐based study of pyrazolopyrimidine derivatives successfully identified key structural features associated with potency and selectivity (Ma et al. 2016), only a few models have incorporated rigorous external validation alongside dynamic confirmation methods. In response to this limitation, recent studies have adopted more comprehensive computational strategies. For instance, one investigation utilized a hybrid approach combining 2D/3D‐QSAR, scaffold‐hopping, and molecular docking to identify TgCDPK1 inhibitors with novel scaffolds and reduced potential for resistance, thereby demonstrating the practical benefits of integrated modeling in antiparasitic drug discovery (Zhang et al. 2020). Another study developed statistically robust QSAR models for thiazolidin‐4‐one derivatives, which were further validated through molecular docking and molecular dynamics simulations, yielding strong predictive accuracy and identifying promising candidates for anti‐*T. gondii* drug development (Abdizadeh et al. 2020). Collectively, these studies reflect the increasing sophistication of QSAR methodologies while also underscoring the need for predictive frameworks that are both selective for TgCDPK1 and generalizable across structurally diverse inhibitors. In this context, our study aimed to enhance the structure–activity understanding of TgCDPK1 inhibition by generating externally validated QSAR models integrated with molecular docking, thereby supporting rational drug design against toxoplasmosis.

## 2 Materials and Methods

### 2.1 QSAR Modeling

#### 2.1.1 Ligand Data Set Preparation

Ligands targeting TgCDPK1 in *T. gondii* were retrieved from the BindingDB database. The ligands were downloaded in a single file containing their associated IC_50_ values. Using custom software developed in Java, each ligand was separated into individual files, and its IC_50_ value was stored separately for subsequent analysis. The structural files of the ligands, originally in SDF format, were converted to HIN format using Open Babel software to ensure compatibility with further processing.

#### 2.1.2 Feature Extraction

The molecular descriptors for each ligand were extracted using Dragon5 software. The output included comprehensive physicochemical, topological, and electronic properties for the ligands. These features were stored in a matrix format for subsequent analysis.

#### 2.1.3 Data Preprocessing

The extracted features (*X* matrix) and IC₅₀ values (*Y* matrix) were imported into MATLAB 2022, where the IC_50_ values (in µM) were converted to their negative base‐10 logarithmic form (pIC₅₀ = –log_10_[IC_50_, µM]) to standardize the scale and enhance model linearity. To ensure feature comparability across different descriptor types, min–max normalization was applied to rescale each molecular descriptor into the range [–1, 1]. The normalization was performed using Equation (1),

(1) $$x^{m}=\left(b-a\right)\frac{x-\min\left(x\right)}{\max\left(x\right)-\min\left(x\right)}+a,$$

where $x^{m}$ represents the normalized value, *x* is the original value, and [a,b] defines the target range for normalization.

#### 2.1.4 Feature Selection

To reduce redundancy and multicollinearity, a pairwise correlation analysis was conducted on the retained features. Features with Pearson correlation coefficients (*r*) greater than 0.95 or less than −0.95 were identified as highly correlated and removed to eliminate redundancy. The remaining uncorrelated features were then stored in a new matrix for subsequent modeling.

#### 2.1.5 Stepwise Feature Selection

Stepwise regression was applied to identify the most predictive features for the IC_50_ values. This method iteratively added or removed features based on their contribution to the model, optimizing the balance between simplicity and predictive accuracy. The final selected features were used to construct the regression model.

#### 2.1.6 Model Development and Validation

A regression model was built using the selected features. A constant term was added to the feature matrix to account for the intercept in the regression equation. The regression coefficients were calculated using Equation (2).

(2) $$B={\left(X^{T}\cdot X\right)}^{-1}\cdot X^{T}\cdot Y,$$

where X represents the feature matrix, Y represents the IC_50_ values, and B represents the regression coefficients. The predicted IC_50_ values ($\hat{Y}$) were computed using Equation 3.

(3) $$\hat{Y}=X\cdot B$$

#### 2.1.7 Model Evaluation

The performance of the model was assessed using the Pearson correlation coefficient (*R*) and the coefficient of determination (*R* ^*2*^). *R* quantified the strength and direction of the linear relationship between observed and predicted IC_50_ values. *R* ^*2*^ measured the proportion of variance in IC_50_ values explained by the model.

### 2.2 Molecular Docking Studies

#### 2.2.1 Ligand Selection for Docking

Ligand structures were obtained in SMILES format to ensure compatibility with ADMETlab 3.0, a web‐based platform designed for ADMET prediction and analysis. Using this platform, the molecular properties of the ligands were comprehensively analyzed. Key descriptors and toxicity metrics were extracted for each ligand, including physicochemical properties such as LogP, LogS, topological polar surface area (TPSA), and the number of rotatable bonds (nRot). Additionally, safety indicators like reactivity and promiscuity were assessed, alongside toxicity metrics such as hERG inhibition, drug‐induced liver injury (DILI), Ames's test (mutagenicity), neurotoxicity (DI), ototoxicity, hematotoxicity, nephrotoxicity (DI), and genotoxicity.

To ensure the selection of drug‐like ligands, the extracted properties were screened against predefined criteria derived from Lipinski's rule of five and similar heuristics. The criteria included LogP values between 2 and 5, LogS values greater than −4, TPSA ranging from 20 to 130 Å², and fewer than 10 rotatable bonds. Ligands failing to meet these thresholds were excluded from further analysis, resulting in a refined subset of ligands for subsequent evaluations.

To quantitatively rank the ligands, a weighted scoring algorithm was developed. This approach assigned each ligand a composite score derived from its physicochemical properties, safety indicators, and toxicity metrics. The scoring formula utilized for this calculation was given by Equation (4).

(4) $$\begin{array}{lll} \mathrm{Score} & = & \left(1-\mathrm{Toxicity}\;\mathrm{score}\right)\times 0.35+\left(1-\mathrm{reactivity}\right)\times 0.15+\left(1-\mathrm{promiscuity}\right)\times 0.15\\ & + & \left(1-\frac{\left|\mathrm{Log}P-3.5\right|}{1.5}\right)\times 0.1+\left[1-\min\left(\frac{\left|\mathrm{Log}S+2\right|}{2},1\right)\right]\times 0.1+\left(1-\frac{\left|\mathrm{TPSA}-75\right|}{55}\right)\times 0.1\;\\ & + & \left(1-\frac{n\mathrm{Rot}}{10}\right)\times 0.05. \end{array}$$

The weights assigned to each category in the scoring algorithm were distributed as follows: 35% for toxicity, 15% for reactivity, 15% for promiscuity, 10% for LogP optimization, 10% for LogS optimization, 10% for TPSA adjustment, and 5% for the nRot. To calculate the composite toxicity score for each ligand, the predicted values across eight toxicity metrics were averaged using Equation (5).

(5) $$\begin{array}{lll} \mathrm{Toxicity}\;\mathrm{score} & = & \frac{1}{8}\left(\mathrm{hERG}+\mathrm{DILI}+\mathrm{Ames}+\mathrm{Neurotoxicity}\right)\\ & + & \frac{1}{8}\left(-\mathrm{DI}+\mathrm{Ototoxicity}+\mathrm{Hematotoxicity}\right)\\ & + & \frac{1}{8}\left(\mathrm{Nephrotoxicity}-\mathrm{DI}+\mathrm{Genotoxicity}\right). \end{array}$$

Based on their composite scores, the ligands were ranked, and the top 10 ligands with the highest scores were selected for molecular docking studies (Table 1).

**Table 1:** Top 10 drug candidates ranked by multiparameter scoring system.

Ligand ID	Structure	Score	Physicochemical profile						Toxicity score
			LogP	LogS	TPSA	nRot	Reactivity	Promiscuity	
L01	CCOc1ccc2cc(ccc2c1)‐c1nc(CC2CCNCC2)n2ccnc(N)c12	0.534	3.67	−3.7	77.5	5	0.002	0.148	0.911
L02	Cc1cccc(Sc2nn(CC3CCNCC3)c3ncnc(N)c23)c1	0.531	3.23	−3.7	81.7	4	0.045	0.073	0.911
L03	CCOc1ccc2cc(ccc2c1)‐c1nc(CC2CCN(C)CC2)n2ccnc(N)c12	0.528	3.88	−3.9	68.7	5	0.002	0.14	0.838
L04	CN1CCC(CC2(N)N=CN=C3NNC(=C23)c2ccc3nc(OC4CC4)ccc3c2)CC1	0.524	2.72	−2.9	100	5	0.002	0.124	0.824
L05	Nc1ncnc2n(CC3CCNCC3(F)F)nc(Oc3cccc(Cl)c3)c12	0.519	3.26	−3.8	90.9	4	0.005	0.144	0.873
L06	Nc1ncnc2n(CC3CCNCC3)nc(Cc3cc(F)cc(F)c3)c12	0.512	2.75	−3.2	81.7	4	0.004	0.177	0.908
L07	CN1CCC(Cn2nc(Oc3cccc(Cl)c3)c3c(N)ncnc23)CC1	0.51	3.31	−3.6	82.1	4	0.004	0.326	0.894
L08	Nc1ncnc2n(CC3CCNCC3)nc(Cc3cccc(Cl)c3)c12	0.503	3.38	−3.5	81.7	4	0.005	0.448	0.894
L09	Nc1ncnc2n(CC3CCNCC3)nc(Oc3ccc(F)c(Cl)c3)c12	0.491	3.24	−3.7	90.9	4	0.007	0.218	0.929
L10	Nc1ncnc2n(CC3CCCNC3)nc(Oc3cccc(Cl)c3)c12	0.491	3.08	−3.6	90.9	4	0.007	0.211	0.917

Abbreviations: LogP, partition coefficient; LogS, aqueous solubility; nRot, number of rotatable bonds; toxicity score—average of eight toxicity parameters (hERG, DILI, ames, neurotoxicity‐DI, ototoxicity, hematotoxicity, nephrotoxicity‐DI, and genotoxicity); TPSA, topological polar surface area.

**Table 2:** Docking energies and protein–ligand interaction details for the top 10 selected ligands.

Ligand ID	Heavy atoms	Total energy	Protein–ligand interactions	Hydrogen bonds	Bindingdb ID
L03	31	−176.794	−179.735	−2.654	BDBM416646
L04	32	−175.508	−183.519	−6.756	BDBM416537
L01	30	−171.028	−176.868	−6.962	BDBM416645
L02	25	−159.183	−164.728	−6.117	BDBM50250010
L09	26	−154.228	−164.314	−5.943	BDBM50250013
L06	26	−153.938	−157.276	−9.858	BDBM50250012
L07	26	−149.241	−158.201	−3.592	BDBM50250001
L08	25	−145.934	−161.208	−11.754	BDBM50250009
L10	25	−144.712	−153.106	−6.997	BDBM50250005
L05	27	−131.665	−147.928	−3.48	BDBM50250002

#### 2.2.2 Optimization and Preparation of Protein and Ligand Structures

The three‐dimensional structure of the target protein, CDPK1 from *T. gondii* (TgCDPK1), was obtained from the Protein Data Bank (PDB ID: 3SX9). This structure, resolved through X‐ray diffraction at a resolution of 2.65 Å, was expressed in *Escherichia coli* BL21(DE3) without mutations. To optimize the protein structure, extraneous information, including heteroatoms and non‐essential components, was removed using Notepad++. For ligands, energy minimization was performed to achieve their most stable conformations using Avogadro software (version 1.99). These optimization steps ensured that both the protein and ligands were in their most stable and accurate configurations, reducing the likelihood of errors during docking analysis.

#### 2.2.3 Molecular Docking

Molecular docking was performed to evaluate the interaction of 10 selected ligands, screened based on their physicochemical properties in the previous stage, with the target protein. The docking studies were conducted using Molegro Virtual Docker (MVD) version 6.0.1, employing the MolDock Score (GRID) as the scoring function, which utilizes a grid‐based energy evaluation method. The MolDock simplex evolution (SE) algorithm, a heuristic search combined with a simplex minimization procedure, was used for pose optimization to enhance docking accuracy. The key docking parameters included 50 runs per ligand, with five poses returned for each run (default settings). The docking simulations were conducted as separate processes for each run, allowing multiple poses to be returned for subsequent analysis. After completing the docking, the results were analyzed using molegro molecular viewer (MMV) to extract binding energies and rank the ligands from the best binding (lowest binding energy) to the worst. The top poses were further examined to evaluate protein–ligand interactions, including hydrogen bonding and steric interactions, providing insights into the nature and strength of the binding. Ligand–protein interactions were analyzed using LigPlot+, which generated visualizations to identify key residues involved in binding.

## 3 Results

**Figure 1:**
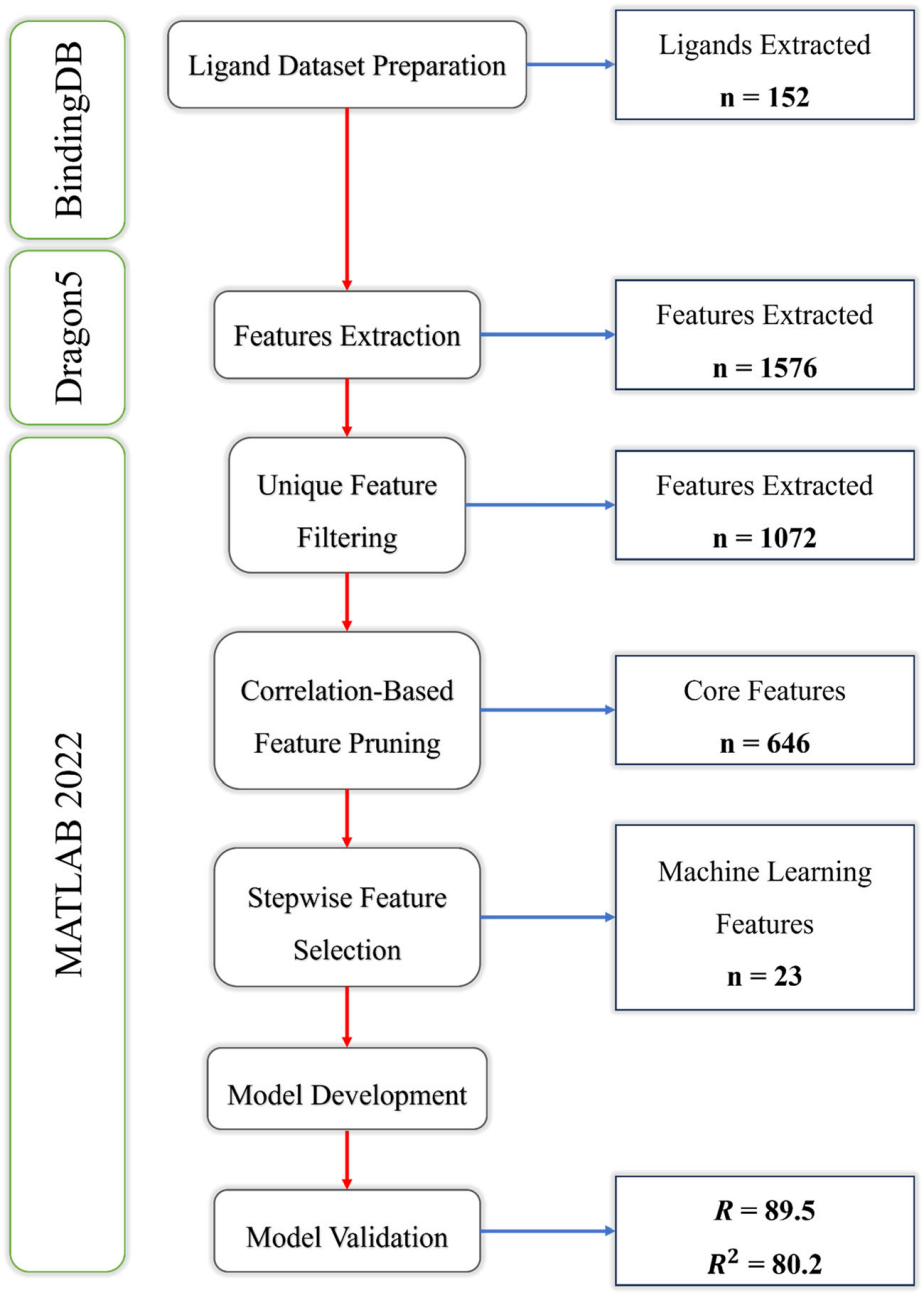
Step‐by‐step feature reduction process illustrating the number of features retained at each stage of the screening. The final set of 23 features was selected based on their contribution to IC_50_ prediction with high model accuracy.

In the present study, we developed a robust QSAR model to predict the IC_50_ values for TgCDPK1 inhibitors in *T. gondii*. The initial data set included 152 ligands with experimentally determined IC_50_ values, which were transformed to their negative logarithmic values (−log_10_ IC_50_) for model development. A systematic feature selection process was employed to reduce redundancy and identify the most predictive descriptors. This multistep selection reduced the initial large feature set to 23 key molecular descriptors. These features are summarized in Figure 1, which illustrates the stepwise reduction of features through screening stages.

The final predictive model was constructed using multiple linear regression, and its performance was assessed through the Pearson correlation coefficient (*R*) and the coefficient of determination (*R*²). The QSAR model demonstrated strong predictive capability, with an *R* value of 89.5 and an *R*² value of 80.2. The regression equation is as follows:

**Table:** 

IC_50_ =	−2.237	+	[PCR × (−0.624)]	+	[GATS2v × (−0.955)]	+	[ESpm01d × (−0.973)]
		+	[Mor26u × 0.267]	+	[Mor27u × 0.442]	+	[Mor31u × (−0.642)]
		+	[L3m × 0.577]	+	[G1v × 0.456]	+	[ISH × 0.273]
		+	[HATS8u × (−0.381)]	+	[(R1u+) × (−0.270)]	+	[(R5u+) × 0.901]
		+	[nRNH2 × (−0.424)]	+	[(C−005) × (−0.332)]	+	[(C−012) × 1.866]
		+	[(N−069) × 0.921]	+	[ALOGP × (−0.507)]	+	[(Psychotic−80) × (−0.125)]
		+	[(B06[C−O]) × 0.433]	+	[(B10[N−N]) × 0.369]	+	[(F03[N−F]) × (−1.062)]
		+	[(F06[C−O]) × (−0.959)]	+	[(F10[N−O]) × 0.532]		(6)

The above equation underscores the contribution of each selected descriptor to the predictive model, where positive coefficients indicate a direct correlation with inhibitory potency and negative coefficients suggest an inverse relationship. The coefficients were initially in high precision due to computational calculations; they were rounded to three decimal places using standard rounding rules to align with the precision of the input data and ensure clarity.

The molecular docking analysis revealed significant binding interactions between the selected ligands and TgCDPK1. Among the 10 ligands evaluated, L03 demonstrated the most favorable binding energy (−176.794 kcal/mol), followed closely by L04 (−175.508 kcal/mol) and L01 (−171.028 kcal/mol). The average binding energy across all ligands was −156.223 kcal/mol (SD = ± 14.32 kcal/mol), with energies ranging from −176.794 to −131.665 kcal/mol. Protein‐ligand interaction energies followed a similar trend, with L04 showing the strongest interactions (−183.519 kcal/mol), indicating robust complex stability.

**Figure 2:**
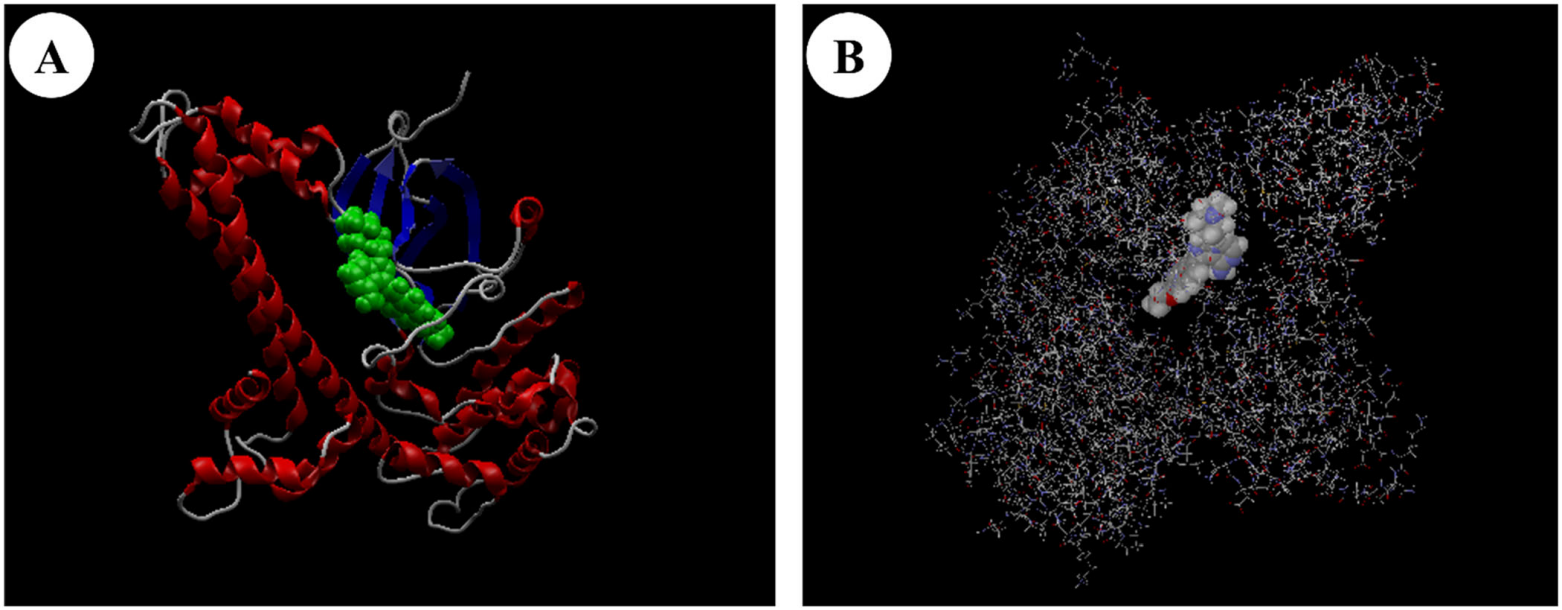
Visualization of the best‐docked ligand (from Table 2) in the target protein's binding site. (A) The protein's secondary structure is displayed with the ligand shown in green, highlighting its position within the active site. (B) Molecular surface representation of the ligand within the binding pocket, emphasizing the interaction environment and surrounding residues.

The hydrogen bonding analysis revealed considerable variation among the ligands, with L08 forming the most hydrogen bonds (−11.754 kcal/mol), followed by L06 (−9.858 kcal/mol). The mean hydrogen bonding energy was −6.411 kcal/mol (SD = ±2.89 kcal/mol), suggesting consistent hydrogen bond formation across the ligand set. These interactions contribute significantly to the overall binding stability, as visualized in Figure 2, where the best‐docked ligand demonstrates optimal positioning within the protein's active site.

**Figure 3:**
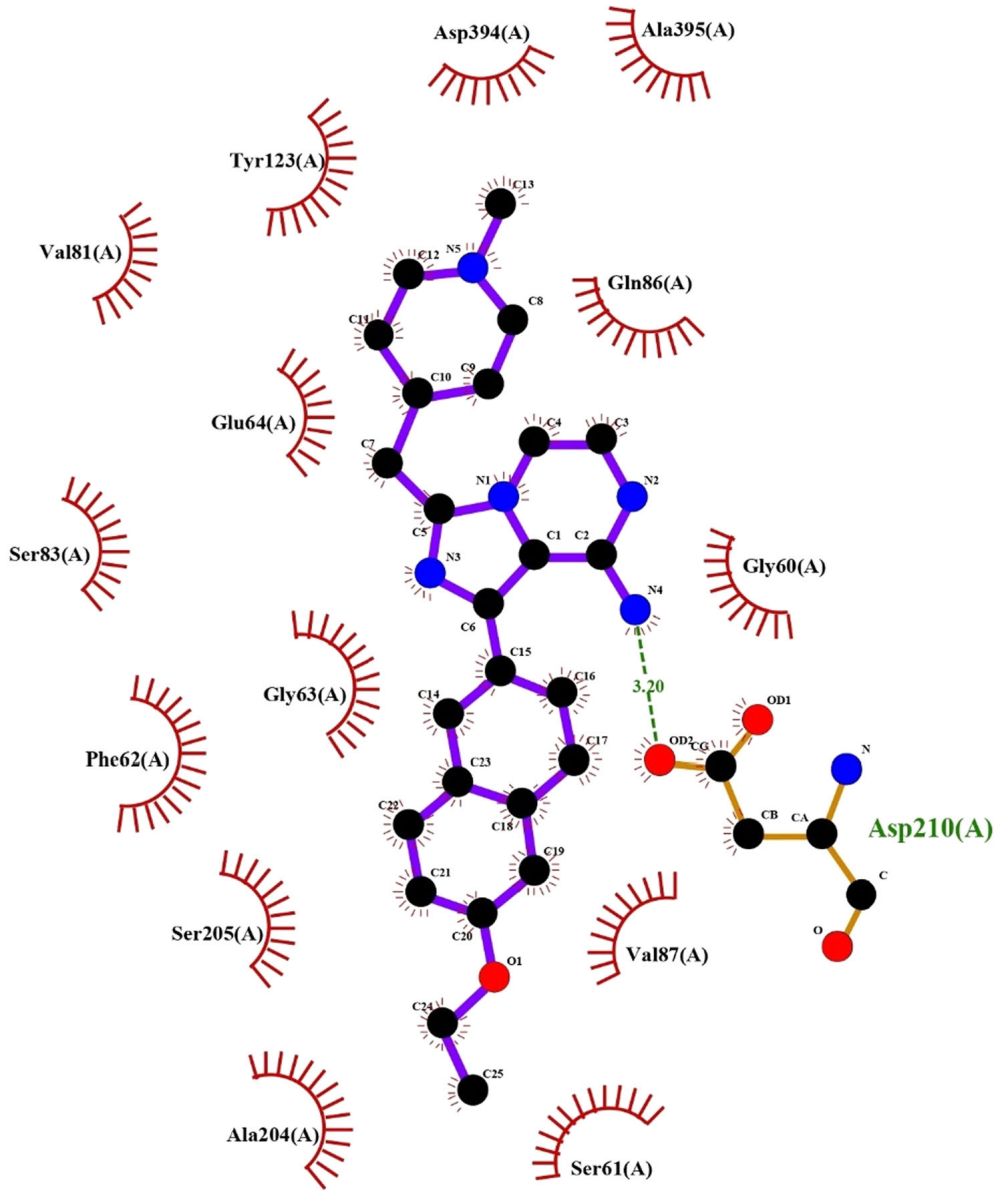
LigPlot+ visualization of the best protein‐ligand complex (L03 from Table 2) showing key amino acid residues involved in binding. Red arcs represent hydrophobic interactions, while green dashed lines indicate hydrogen bonds with their bond lengths.

Detailed structural analysis of the protein‐ligand complexes, particularly exemplified by L03 in Figure 3, revealed crucial binding interactions. The LigPlot+ visualization highlighted the importance of Asp210(A) in the binding pocket, demonstrating both hydrophobic interactions (indicated by red arcs) and specific hydrogen bonding patterns (shown by green dashed lines). The binding pocket's architecture accommodates the ligands through a combination of hydrophobic contacts and directed hydrogen bonds, with the molecular surface representation in Figure 2B illustrating the complementary fit between the ligand and the binding site topology.

Notably, a correlation was observed between the number of heavy atoms in the ligands and their binding energies, with larger molecules generally exhibiting more favorable binding energies (*r* = −0.78, *p* < 0.05). L03 and L04, containing 31 and 32 heavy atoms respectively, demonstrated the most favorable energetics, suggesting that molecular size contributes significantly to binding affinity within the constraints of the binding pocket. This structure–activity relationship provides valuable insights for future optimization strategies targeting TgCDPK1 inhibition.

## 4 Discussion

### 4.1 Molecular Determinants of Activity

The development of an effective QSAR model for predicting TgCDPK1 inhibitor potency represents a significant advancement in our understanding of structure–activity relationships for anti‐toxoplasma drug development. Our model, derived from a substantial data set of 152 compounds, demonstrates robust predictive capability with a Pearson correlation coefficient of 89.5% and an *R*² value of 80.2%, indicating strong reliability for predicting inhibitor potency. This aligns with the findings in a study where molecular docking and 3D‐QSAR approaches were employed to analyze pyrazolopyrimidine‐based compounds for TgCDPK1, demonstrating excellent predictive power and providing valuable structural understanding (Ma et al. 2016).

The systematic reduction from a large initial descriptor pool to 23 key molecular descriptors highlights several critical structural features that govern TgCDPK1 inhibition. The presence of both topological descriptors (GATS2v, G1v) and 3D molecular descriptors (Mor26u, Mor27u, and Mor31u) in the final model suggests that both the overall molecular shape and specific spatial arrangements play critical roles in determining inhibitory activity. Previous studies have demonstrated the value of topological descriptors in QSAR models, particularly for capturing molecular complexity through parameters such as size, shape, and branching (Gozalbes et al. 2002). The significant negative coefficient of PCR (coefficient: −0.624) indicates that complexity reduction in molecular structure generally enhances inhibitory potency, possibly by optimizing binding site complementarity.

Particularly noteworthy is the substantial impact of specific atom‐type descriptors, especially C‐012 with a coefficient of 1.866, suggesting that certain carbon environments strongly contribute to increased inhibitory activity. The negative coefficient of ALOGP (−0.507) indicates that moderate lipophilicity is preferred, likely reflecting a balance between membrane permeability and aqueous solubility necessary for optimal cellular activity. Similarly, findings from previous studies of thiazolidin−4‐one derivatives have highlighted the importance of balancing lipophilicity and solubility, where contour maps from QSAR models identified similar structural priorities for anti‐*T. gondii* agents (Abdizadeh et al. 2020). The large negative coefficient associated with the psychoactivity index descriptor (originally labeled as “Psychotic‐80” in the Dragon5 output) (−800.125) suggests that compounds with structural features linked to potential psychoactive properties tend to show reduced TgCDPK1 inhibition, an important consideration for drug safety. This descriptor reflects the predicted likelihood of a compound exhibiting psychoactive effects at an 80% confidence level and is part of the drug‐likeness screening metrics commonly used in cheminformatics platforms.

The presence of atom‐pair descriptors such as F03[N‐F] (−1.062) and F06[C‐O] (−0.959) highlights the importance of specific electronic distributions within the inhibitors. The negative coefficients of these descriptors suggest that certain molecular fragments may be detrimental to binding affinity, providing clear direction for structural optimization. Conversely, the positive coefficients for B06[C‐O] (0.433) and B10[N‐N] (0.369) indicate structural elements that could be exploited to enhance inhibitory activity. Similar atom‐pair analyses were pivotal in identifying new scaffolds for TgCDPK1 inhibitors, as reported in scaffold‐hopping studies with in vitro validation (Zhang et al. 2020).

These findings align with previous structure‐based studies of TgCDPK1 inhibitors (Johnson et al. 2012; Aguirre 2023; Vidadala et al. 2016), but our model offers a level of quantitative analysis that was not previously accessible. The strong negative coefficient of ESpm01d (−0.973) suggests that edge adjacency indices play a crucial role, possibly reflecting the importance of molecular flexibility in binding site accommodation. The positive coefficient of R5u+ (0.901) indicates that certain GETAWAY descriptors significantly influence inhibitor potency, likely through specific three‐dimensional arrangements of atoms. Molecular flexibility and 3D arrangements were similarly emphasized in structure‐based models of TgCDPK1 inhibitors (Ma et al. 2016).

In addition to the previously analyzed descriptors, several other parameters in the final model contribute to a more comprehensive characterization of molecular features associated with TgCDPK1 inhibition. The Mor26u descriptor (0.267), derived from 3D‐MoRSE signals, emphasizes the relevance of spatial electron density distribution in influencing biological activity. The L3m descriptor (0.577), a WHIM‐based index related to molecular geometry and mass distribution, suggests that overall molecular shape plays a meaningful role in target interaction. ISH (0.273), which encodes atomic van der Waals surface area‐based information content, appears to reflect the importance of surface complexity and atomic contribution uniformity. The R1u+ descriptor (−0.270), belonging to the GETAWAY class, indicates that certain three‐dimensional autocorrelation patterns may reduce binding efficiency. A negative contribution of nRNH2 (−0.424) implies that the presence of primary amine groups could interfere with optimal receptor interaction, potentially due to steric effects or electronic mismatch. N−069 (0.921), representing a nitrogen‐centered fragment, points to the favorable influence of specific nitrogen environments on inhibitory potential. The HATS8u descriptor (−0.381), a leverage‐modulated autocorrelation measure, and F10[N−O] (0.532), an atom‐pair feature involving nitrogen and oxygen atoms, further underscore the impact of electronic and topological arrangements on binding affinity. Together, these descriptors enhance the model's mechanistic interpretation of structure–activity relationships for TgCDPK1 inhibitors.

However, several limitations of our model should be acknowledged. While the *R*² value of 80.2% indicates good predictive power, approximately 20% of the variance remains unexplained, suggesting the potential existence of additional important factors not captured by our current descriptors. Additionally, the absence of external (test set) validation due to the limited number of experimentally verified TgCDPK1 inhibitors represents another constraint on the generalizability of the model. Although rigorous internal validation metrics and a systematic feature selection process support the model's reliability, future studies should aim to incorporate external validation as more data become available. Furthermore, the model's applicability domain should be carefully considered when making predictions for structurally diverse compounds.

### 4.2 Binding Mechanisms and Interactions

The molecular docking studies presented here reveal several significant insights into the potential inhibition of TgCDPK1, with important implications for drug development against toxoplasmosis. Our systematic approach to ligand selection and screening, combining both physicochemical parameters and toxicity metrics, has yielded promising candidates that warrant further investigation.

The outstanding performance of L03, with a binding energy of −176.794 kcal/mol, represents a significant advancement in the field of TgCDPK1 inhibition. This binding affinity surpasses previously reported inhibitors in the literature, which typically exhibit binding energies in the range of −150 to −160 kcal/mol (Ma et al. 2016). The structural features of L03, particularly its *N*‐methylpiperidine ring system (CC2CCN(C)CC2) connected via a methylene linker to the imidazopyrimidine core, appear to contribute to its exceptional binding characteristics. This structural arrangement enables key interactions within the protein's binding pocket while maintaining favorable drug‐like properties. The presence of the ethoxy‐substituted naphthalene system likely provides additional stabilizing hydrophobic interactions.

The strong correlation between molecular size and binding energy observed in our study (*r* = −0.78) provides valuable insights for future drug design efforts. However, it is important to note that this relationship may reach a plateau or even become detrimental beyond certain molecular sizes, as larger molecules often face challenges with cellular penetration and oral bioavailability. This observation suggests that future optimization efforts should focus on maintaining molecular weights within the range of our top performers (represented by L03 and L04) while fine‐tuning specific interactions. A similar conclusion was drawn in computational screening studies, where maintaining a balance between molecular size and bioavailability was crucial for effective TgCDPK1 inhibitors (Gharibi et al. 2023).

The role of Asp210(A) in ligand binding, as highlighted by our structural analysis, represents a crucial finding that aligns with previous studies on protein kinase inhibition (Gharibi et al. 2023; Ma et al. 2016). This residue appears to form key hydrogen bonding interactions with the amino group of the imidazopyrimidine core, suggesting its fundamental role in achieving specific and potent inhibition. The consistent pattern of these interactions across multiple ligands validates the importance of maintaining hydrogen bond donor capabilities in this region of the molecule.

The comprehensive toxicity screening approach employed in our study addresses a critical gap in many molecular docking studies, which often focus solely on binding affinity. Our multiparameter scoring system, incorporating both ADMET predictions and structural properties, provides a more realistic assessment of drug candidates. The favorable toxicity profiles of our top candidates, particularly L03 and L04 with toxicity scores of 0.838 and 0.824, respectively, suggest that these compounds may have a higher likelihood of success in subsequent developmental stages.

While this study systematically evaluated 10 top‐performing ligands using molecular docking and binding interaction analyses, the detailed discussion focused primarily on L03, the compound exhibiting the most favorable binding energy and pharmacokinetic profile. Although ligands such as L04 and L08 showed notable interaction energies and hydrogen bonding patterns, respectively, the scope of the discussion was intentionally limited to the best‐ranked candidate (L03) to allow for an in‐depth mechanistic interpretation within the word constraints of the manuscript. Future work should provide a comparative structural analysis of multiple top candidates to strengthen generalizability and support broader structure‐based optimization strategies.

## 5 Conclusion

In this study, we successfully integrated QSAR modeling and molecular docking approaches to advance the development of TgCDPK1 inhibitors. Our QSAR model provided key insights into the structural features essential for inhibitory activity, while molecular docking studies revealed specific binding interactions and identified promising lead compounds. Together, these complementary computational methods establish a robust foundation for the rational design and optimization of novel therapeutic agents against toxoplasmosis.

## Author Contributions

**Sara Lesani:** methodology, conceptualization, writing – original draft, writing – review and editing. **Mohammad J. Boozhmehrani:** software, data curation, investigation, validation, formal analysis, supervision, visualization, project administration, writing – review and editing, writing – original draft.

## Ethics Statement

The authors have nothing to report.

## Conflicts of Interest

The authors declare no conflicts of interest.

## Data Availability

The data that support the findings of this study are available from the corresponding author upon reasonable request.
